# Does Vitamin D Supplementation Impact Fibromyalgia-Related Pain? A Systematic Review and Meta-Analysis

**DOI:** 10.3390/nu17203232

**Published:** 2025-10-15

**Authors:** Sara Ilari, Saverio Nucera, Valentina Malafoglia, Stefania Proietti, Lucia Carmela Passacatini, Rosamaria Caminiti, Valeria Mazza, Alessia Bonaddio, Francesca Oppedisano, Jessica Maiuolo, Daniela Caccamo, Marco Tafani, Carlo Tomino, Vincenzo Mollace, William Raffaeli, Carolina Muscoli

**Affiliations:** 1 Istituto di Ricovero e Cura a Carattere Scientifico (IRCCS) San Raffaele Roma, 00166 Rome, Italy; sara.ilari@sanraffaele.it (S.I.); valentina.malafoglia@sanraffaele.it (V.M.); carmela.passacatini@sanraffaele.it (L.C.P.); carlo.tomino@sanraffaele.it (C.T.); 2 Department of Human Sciences and Quality of Life Promotion, San Raffaele University, 00166 Rome, Italy; 3 Department of Health Sciences, Institute of Research for Food Safety and Health (IRC-FSH), University “Magna Graecia” of Catanzaro, 88100 Catanzaro, Italy; rosamaria.caminiti@studenti.unicz.it (R.C.); valeria.mazza001@studenti.unicz.it (V.M.); foppedisano@unicz.it (F.O.); maiuolo@unicz.it (J.M.); mollace@libero.it (V.M.); 4 Agea, Coordinating Body, Office Study Center, 00185 Rome, Italy; 5 Department of Biomedical and Dental Sciences and Morpho-Functional Imaging, University of Messina, 98125 Messina, Italy; daniela.caccamo@unime.it; 6 Department of Experimental Medicine, Sapienza University of Rome, 00161 Rome, Italy; marco.tafani@uniroma1.it; 7 Istituto per la Ricerca sul Dolore, Fondazione ISAL, 47921 Rimini, Italy; isal@fondazioneisal.it

**Keywords:** fibromyalgia, vitamin D supplementation, chronic pain, systematic review, meta-analysis, pain management

## Abstract

**Background:** Fibromyalgia is a chronic condition characterized by widespread pain, fatigue, and localized tenderness. Its pathophysiology remains unclear, and treatment options are often limited and only partially effective. Recent studies suggest a potential link between vitamin D deficiency and symptom severity, as vitamin D may play a role in modulating pain and inflammation. **Methods:** This systematic review and meta-analysis assessed the efficacy of vitamin D supplementation in reducing pain and improving quality of life in fibromyalgia patients, focusing on studies up to 31 December 2024. Following PRISMA guidelines, a literature search in PubMed, Web of Science, and Scopus identified 2776 articles; 7 were included in the systematic review and 4 studies in each meta-analysis. **Results:** Results showed that vitamin D supplementation significantly reduced pain levels compared to the control group, with a statistically significant effect observed using the NRS or VAS (SMD = −0.85; 95% CI: −1.54 to −0.17; *p* = 0.0148), as well as the FIQ scale (SMD = −0.87; 95% CI: −1.56 to −0.20; *p*= 0.0115), resulting in an improvement in quality of life. **Conclusions:** These findings suggest that vitamin D may be a valuable adjunct in fibromyalgia management, particularly for pain. However, further high-quality trials are needed to confirm these effects and identify responsive patient subgroups.

## 1. Introduction

Fibromyalgia syndrome (FMS) is a complex and multifactorial chronic disorder that affects approximately 2–3% of the global population, with a higher prevalence among women [1,2]. Characterized by widespread musculoskeletal pain, generalized fatigue and weakness, sleep and mood disturbances, FMS represents a diagnostic and therapeutic challenge for clinicians [3,4,5].

Chronic pain biomarkers could play a crucial role in understanding the mechanisms underlying the pathology, identifying homogeneous subgroups of patients—including those with fibromyalgia—within heterogeneous clinical conditions, and providing valuable insights into the biological changes associated with therapeutic interventions.” [6,7,8].

The diagnostic criteria established by the American College of Rheumatology (ACR) in 1990, and subsequently updated in 2010, require the presence of persistent symptoms for at least three months, combined with the exclusion of other pathological conditions that could mimic FMS [1,2]. From a pathophysiological point of view, fibromyalgia is often described as a central sensitization syndrome, in which a dysfunction of the neurocircuits involved in the perception, transmission and processing of painful stimuli is observed [9].

Pain, the cardinal symptom of FMS, is typically described as dull, although it can also present in acute, stabbing or burning forms [10].

Pain represents not only physical discomfort, but also a condition with significant social consequences [11].

Although pain is predominantly muscular, many patients also report joint swelling, despite the absence of synovitis or evident structural alterations [12,13]. Common sites of pain include the upper trapezius, lateral epicondyle, second costochondral junction, and greater trochanter, with possible extension to the head, lower back, buttocks, and abdomen [1,14]. Morning stiffness and exacerbation of pain during prolonged inactivity or minimal activity are additional hallmarks [10]. Approximately 25% of FMS patients also have joint hypermobility, a factor that can further complicate pain management [14].

Despite advances in the understanding of FMS, its pathogenesis remains elusive. Recent studies suggest a significant role of neuroendocrine, metabolic and immunological factors, with particular focus on neurotransmitters such as serotonin, melatonin and substance P [15,16]. Additionally, vitamin D deficiency has been associated with fibromyalgia-like symptoms, such as chronic widespread pain and fatigue, although the link between the two remains controversial [9,17]. Interestingly, vitamin D levels have been reported to be generally lower in FMS patients than in healthy controls [18]. Moreover, higher concentrations of pro-inflammatory cytokines have been observed in patients showing a higher impact of fibromyalgia in daily life, as assessed by the Fibromyalgia Impact Questionnaire (FIQ)-revised (FIQ-R) and a lower vitamin D level. In particular, a significant negative correlation has been found between vitamin D concentrations and FIQ-R scores as well as IL-17 levels [19].

Vitamin D, a fat-soluble compound essential for mineral homeostasis and bone health, has recently gained increasing interest in the management of chronic pain and quality of life improvement [20]. Indeed, preliminary evidence suggests a potential therapeutic role in the management of FMS [21,22]. Vitamin D exists in two main forms, ergocalciferol (vitamin D2) from vegetables and cholecalciferol (vitamin D3) from animal foods. Vitamin D precursor is 7-dehydrocholesterol, which is synthesized in the skin by the action of UVB rays, then converted into calcitriol (1,25-dihydroxyvitamin D), the metabolically active form, by two subsequent hydroxylation steps performed in the liver and kidney [22].

Vitamin D acts as a hormone, displaying a pleiotropic modulatory action specifically mediated by the activation of the ubiquitously distributed vitamin D receptor (VDR), which binds to the retinoid receptor in a heterodimer transcriptional complex able to either activate or inhibit the expression of gene targets involved in various biological processes [23]. Vitamin D regulates calcium and phosphate levels in the blood, influencing bone metabolism, immune system activation, neuroprotection, and muscle function [24,25]. Numerous studies reported polymorphisms in the VDR gene and showed that VDR gene variants influence many biological endpoints and play a major role in various clinical conditions [26]. Vitamin D deficiency and/or the presence of VDR polymorphisms have been independently associated with a variety of chronic pathological conditions, such as osteoporosis, rickets, malignancies, cardiovascular disorders, neurological diseases, type II diabetes, autoimmune diseases, various inflammatory disorders, and with increased risk for all-cause mortality in the general population [23,27]. In vitro studies have shown that vitamin D can reduce the synthesis of prostaglandin E2 (PGE2), a key pro-inflammatory mediator, through downregulation of inflammatory pathways [28]. The anti-inflammatory effects of vitamin D have also been attributed to its impact on peripheral blood mononuclear cells [29], in particular T cell differentiation and the development of regulatory T cell populations, which modulate the pro-inflammatory responses of Th1 and Th17 cells [30,31].

It is still unknown whether vitamin D modulates N-methyl-D-aspartate (NMDA) receptors, glutamate-gated and ligand-gated calcium channels. However, a study on neurotransmitter receptors revealed a reduced expression of the NMDA receptor subunit after vitamin D administration, which contributes to the protective effect of vitamin D against neurotoxicity [32]. These mechanisms suggest that vitamin D could positively influence neuroplasticity and pain modulation. In fact, vitamin D supplementation has been associated with improved musculoskeletal pain in several populations [33].

These properties have led to numerous clinical studies aimed at evaluating the efficacy of vitamin supplementation in conditions such as FMS and musculoskeletal pain syndromes.

Treatment of fibromyalgia requires a multidisciplinary approach, including lifestyle modifications, medical treatment, nutritional support, and cognitive-behavioral therapies [34]. Serotonin–norepinephrine reuptake inhibitors (SNRIs) and antiepileptic drugs are commonly used for pharmacological treatment, due to their favorable side effect profile [35]. Nonsteroidal anti-inflammatory drugs and opioid analgesics are also used to control symptoms, although their use is often limited by side effects [36].

This systematic review and meta-analysis aim to evaluate the efficacy of vitamin D supplementation in reducing pain and improving quality of life in patients with fibromyalgia, focusing on studies published in the last six years. By synthesizing the most recent evidence, the study aims to provide updated clinical recommendations and identify gaps in the current literature.

## 2. Materials and Methods

### 2.1. Database Sources

The systematic review and meta-analysis include data from published experimental studies examining the effect of vitamin D on the improvement of pain and the impact of FMS on quality of life.

The studies included in the review were selected in accordance with the PRISMA (Preferred Reporting Items for Systematic Reviews and Meta-Analyses) statement and following the PICOS framework (Population, Intervention, Comparison, Outcome) (Table 1) [37]. The protocol is registered by Prospero n. CRD420251052916.

A comprehensive literature search was conducted in PubMed/MEDLINE, Scopus, and Web of Science. The search combined controlled vocabulary (MeSH terms in PubMed) and free-text terms (synonyms, spelling variants, and truncations) using Boolean operators (AND, OR).

The PubMed strategy was:

(“Fibromyalgia”[Mesh] OR fibromyalgia[tiab]) AND (“Vitamin D”[Mesh] OR “Cholecalciferol”[Mesh] OR “Ergocalciferols”[Mesh] OR “calcifediol”[tiab] OR “calcitriol”[tiab] OR “vitamin D”[tiab] OR “vitamin D supplementation”[tiab] OR “vitamin D integration”[tiab] OR “use of vitamin D”[tiab] OR “cholecalciferol supplementation”[tiab] OR “cholecalciferol integration [tab]”).

This PubMed strategy was adapted for Scopus and Web of Science, as follows:

Scopus: TITLE-ABS-KEY ((fibromyalgia) AND (“Vitamin D” OR Cholecalciferol OR Ergocalciferols OR calcifediol OR calcitriol OR “vitamin D” OR “vitamin D supplementation” OR “cholecalciferol supplementation” OR “vitamin D integration” OR “cholecalciferol integration”)) AND PUBYEAR > 1993 AND PUBYEAR < 2025

Web of Science: TS = (Fibromyalgia) AND TS = (Vitamin D OR Cholecalciferol OR Ergocalciferol OR Calcifediol OR Calcitriol OR Vitamin D Supplement* OR Cholecalciferol Supplement*).

Only articles published in English were considered. The last search was conducted on 1 July 2025.

Reference lists of all retrieved articles were reviewed to identify additional eligible studies that were not indexed in the above-mentioned databases. Only studies reporting quantitative outcomes relevant to the objectives were included, while articles lacking numerical data or providing incomplete results were excluded. In cases where data were missing or unclear, attempts were made to contact the corresponding authors; however, no additional data were obtained.

A semi-automatic approach using the MySLR platform version 1.0 (https://myslr.unical.it accessed on 1 September 2025) [38], was employed to facilitate the screening and selection process. Specifically, results exported from databases were uploaded to MySLR.

An initial article screening phase was conducted, first eliminating duplicates using the platform’s dedicated functionality. Next, the program’s integrated filters were applied to exclude document types not relevant to the study, including reviews, the literature reviews, narrative reviews, chapters, letters, documents, and books, focusing exclusively on original research articles.

The MySLR platform significantly facilitated the screening phase thanks to its interface optimized for systematic reviews. The clear organization of content and simplified article display allowed for efficient evaluation of all remaining contributions. Each article was analyzed in detail by independent researchers who applied predetermined eligibility criteria to select the studies for inclusion in the final analysis.

This process, enhanced by MySLR’s features, ensured a rigorous and reproducible selection of relevant literature, minimizing study selection bias. The systematic approach adopted, combining automated tools and human assessment, allowed for a comprehensive identification of the available scientific evidence on the research topic [39,40].

### 2.2. Study Selection

Studies were considered eligible if they involved human participants, investigated the effects of vitamin D supplementation. Cross-sectional studies were excluded from the analysis; observational studies were excluded because there were only two, which were insufficient to perform a meta-analysis. Studies were excluded if they involved animal or in vitro experiments, if vitamin D was not included as part of the intervention, or if outcomes were reported exclusively for populations with chronic widespread pain (CWP) without a confirmed fibromyalgia diagnosis, to ensure diagnostic consistency. Additional exclusion criteria included conference abstracts, editorials, book chapters, and studies with incomplete or insufficient data for analysis (as summarized in Table 1).

### 2.3. Data Extraction and Quality Assessment

The main objective of this systematic review and meta-analysis was to assess the pharmacological supplementation of vitamin D in FMSpatients and determine whether such supplementation could help reduce the negative impact of FMs on quality of life.

Based on the data retrieved from the literature search, we conducted two separate meta-analyses.

In the first meta-analysis, we focused on evaluating the analgesic efficacy of vitamin D between subjects in the experimental group (FMS patients treated with vitamin D supplementation) and those in the control group (fibromyalgia patients), using the Numerical Rating Scale (NRS) or Visual Analog Scale (VAS).

In the second meta-analysis, the primary aim was to assess the improvement in the impact of FMS on quality of life following supplementation with vitamin D3 cholecalciferol, using FIQ or FIQ-R. We compared the results between the different groups, including both the experimental group (FMS patients treated with vitamin D supplementation) and the control group (FMS patients).

### 2.4. Statistical Analysis

Continuous outcomes measured on the same scale were described using the mean value and standard deviation in FMS patients, distinguishing between those who took vitamin D (experimental group) and those who did not (control group), in order to obtain summary measures of the effect score in the form of a mean value. For quantitative synthesis, standardized mean differences (SMDs) and their corresponding 95% confidence intervals (95% CI) were calculated. Heterogeneity across studies was assessed using the I^2^ statistic and Cochran’s Q test, following Cochrane Review guidelines. When significant heterogeneity was detected (I^2^ > 50%), a random-effects model was applied [41]. To explore potential contributors to the study variability, univariate meta-regression analyses were conducted to assess the influence of confounding variables on the SMD. The Q-test for moderators (QMs) was used to evaluate the significance of each covariate. Publication bias was assessed using funnel plots, adjusted with the “trim and fill” method, and statistically tested using Egger’s test. All statistical tests were two-tailed, and significance was set at *p* < 0.05 [42].

A meta-regression analysis was carried out to investigate and clarify the heterogeneity observed in the findings. The model incorporated gender distribution, age, treatment duration (in weeks) and Vitamin D dosage as explanatory variables.

Finally, to evaluate the robustness of the estimates measures, a sensitivity analysis was performed using the leave-one-out method, implemented with the metainf command. All analyses were carried out using R software (version 3.6.2) with the metafor and meta packages.

## 3. Results

### 3.1. Data Collection

After a thorough screening phase, 7 articles were selected that evaluated the effect of vitamin D supplementation on the improvement of pain and quality of life in patients with FMS. The systematic review initially identified 2776 records from the literature search (Pubmed n = 119; Web of Science n = 204; Scopus n = 2453).

After eliminating 364 duplicates, 2412 articles were examined, among which 191 were excluded because they consisted of review, the literature review, narrative review, chapters, letters, documents, books or conference abstracts.

Of the 794 remaining articles, 787 were discarded because they did not meet the inclusion criteria: they were non-original studies, paid articles, publications in languages other than English, research that included pediatric population, in vivo or in vitro studies, cross-sectional studies or reported outcomes exclusively in populations CWP, without a specific diagnosis of FMS.

At the end of the process, 7 articles were included in the qualitative analysis (systematic review). Two observational studies were excluded because they were insufficient to conduct a meta-analysis, leaving 5 articles for the quantitative analysis (meta-analysis), which was divided into NRS and FIQ analyses. Each meta-analysis included four articles.

**Figure 1:**
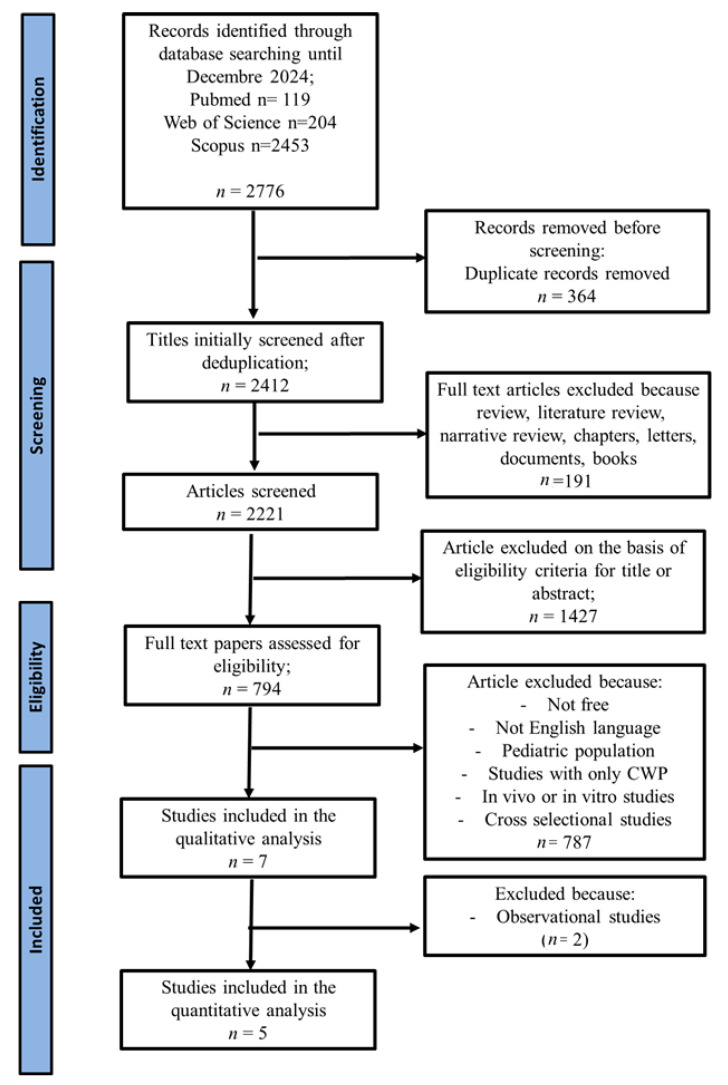
PRISMA flow diagram illustrating the selection algorithm for eligible studies [37].

The literature search and screening process is detailed in Figure 1 [37].

### 3.2. Characteristics of the Studies Included

Table 2 summarizes the characteristics of studies reporting NRS or VAS and FIQ values. Articles in the table appear in decreasing order by year of publication.

Among the most used tools to evaluate the effectiveness of interventions, we find the NRS for the quantification of pain intensity and the FIQ for the multidimensional assessment of quality of life [43]. A critical analysis of the most recent studies, conducted up to 2024, reveals a complex and sometimes contradictory picture, which deserves a thorough examination.

A study conducted by Lozano-Plaza et al. in 2021, including 80 patients, showed no statistically significant differences in FIQ scores between the treated and control groups, neither at baseline nor final assessment, nor in the delta FIQ analysis. Interestingly, although the increase in serum vitamin D levels in the intervention group was clearly documented, this did not translate into measurable clinical improvement [44].

The strongest evidence highlighting the beneficial effects of vitamin D supplementation was provided by a study showing a progressive improvement in both NRS and FIQ scores during the rehabilitation of FMS patients, with apparent benefits after three months of treatment and further significant improvements after six months. Of particular interest is the observation that high-dose vitamin D supplementation demonstrated a dual temporal effect: reduction in musculoskeletal pain in the short term (3 months) and improvement of functional capacity in the long term (6 months) [9]. These benefits were particularly marked in the elderly population, while improvements were less evident in younger patients, suggesting a possible influence of age on the response to treatment.

Similar results were also observed in the studies by Wepner (2016) and Yilmaz (2014) [45,46]. In particular, vitamin D supplementation in patients with FMS and low serum 25(OH)D levels led to a significant improvement in symptoms, especially pain and quality of life. A significant reduction in FIQ (Fibromyalgia Impact Questionnaire) scores was observed in the vitamin D group compared to the placebo group.

Comparable findings were also reported by Mirzaei et al. (2018), who noted an improvement in the quality of life of FMS patients following vitamin D supplementation. Similarly, Dogru et al. (2017) found improvements in physical function, physical and emotional role limitations, social functioning, mental health, vitality, and overall quality of life after treatment with vitamin D [47,48].

This chronological analysis of the most recent studies reveals a complex and multifaceted scenario. On the one hand, studies such as that of Scaturro and coworkers (2022) [9] provide evidence supporting a beneficial role of vitamin D, particularly in the short to medium term and in specific subpopulations, such as the elderly.

On the other hand, studies such as those, Lozano-Plata and coworkers (2021) [44], suggest that the benefits may be more limited or influenced by confounding variables.

This discrepancy could be explained by several methodological and clinical factors: differences in supplementation protocols (dosages, duration of treatment), variability in the characteristics of the populations studied (age, comorbidities, baseline vitamin D levels), heterogeneity in the painful conditions assessed, and differences in outcome measurement tools. Furthermore, the possible role of individual factors (genetic, metabolic, environmental) that could influence the response to treatment should not be overlooked.

**Table 2:** General characteristics of the included studies.

Study/Year	Sex	Age Mean	Number of Patients	Vitamin D Dosage	Duration of Treatment	Assessment Tool	Results
Scaturro et al., 2022 [9]	56 F, 4 M	Age (years): 58.3 ± 4.8	60	25,000 IU	24 weeks	NRS and FIQ	Improvement of NRS and vitamin D levels at both T1 and T2, improvement in FIQ at T1, but no major differences emerged at T2.
Scaturro et al., 2022 [9]	18 F, 2 M;	Age (years): 41.2 ± 6.1	20	25,000 IU	24 weeks	NRS and FIQ	Improvement in musculoskeletal pain and long-term quality of life also emerged.
Lozano-Plata et al., 2021 [44]	F	Age (years): 50.3 ± 11.9	40	50,000 IU	12 weeks	VAS and FIQ	No significant differences emerged in the FIQ score between the various vitamin D subgroups.
Mirzaei et al., 2018 [47]	F	Age (years) 42.1 ± 10.8	37	50,000 IU	8 weeks	FIQ	Improvement in the FIQ score in patientrs with vitamin D supplementation
Dogru et al., 2017 [48]	F	Age (years) 38.7 ± 5.2	42	50,000 IU	12 weeks	VAS and FIQ	Improvement of pain and FIQ score in patients wirn vitamin D supplementation
Yilmaz et al., 2016 [46]	52 F, 6 M	Age (years) 36.9 ± 9.2	58	50,000 IU	12 weeks	VAS	Improvement of pain in patients wirn vitamin D supplementation
Wepner et al., 2014 [45]	27 F, 3 M	Age (years) 49.1 ± 5.7	15	2400 IU	49 weeks	VAS and FIQ	Improvement of pain and FIQ score in patients wirn vitamin D supplementation

F: female; M: male; FIQ: Fibromyalgia Impact Questionnaire; NRS: Numerical Rating Scale; VAS: Visual Analog Scale, and IU: International Unit.

### 3.3. Meta-Analysis

The systematic review identified a total of seven studies, divided into two meta-analysis subgroups based on research reporting NRS/VAS and FIQ values. Each meta-analysis included four articles. Subjects of both sexes were considered.

### 3.4. Meta-Analysis and NRS/VAS

Studies included in this meta-analysis assessed the analgesic efficacy of vitamin D in both the experimental group (patients with FMS treated with vitamin D supplementation) and the control group (FMS patients), using the NRS or VAS.

In this first meta-analysis, a total of 155 subjects were included in the experimental group and 155 in the control group.

**Figure 2:**
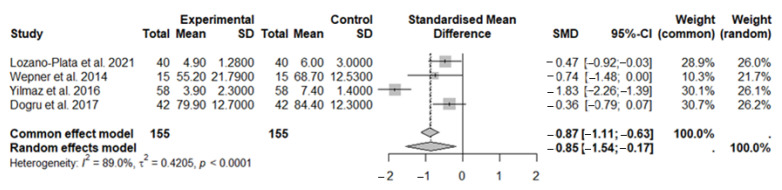
The forest plot identifies the analgesic effect of vitamin D in FMS patients, measured using the NRS/VAS. SD, standard deviation; SMD, standard mean difference; CI, confidence interval [44,45,46,48].

Results obtained using the random-effects model demonstrated a significant beneficial effect of vitamin D supplementation on fibromyalgia syndrome compared to the control group (SMD: −0.85; 95% CI: −1.54 to −0.17; *p* = 0.0148), contributing to pain reduction. A statistically significant high level of heterogeneity was observed among the studies: I^2^ = 89%, *p* < 0.0001 (Figure 2).

**Figure 3:**
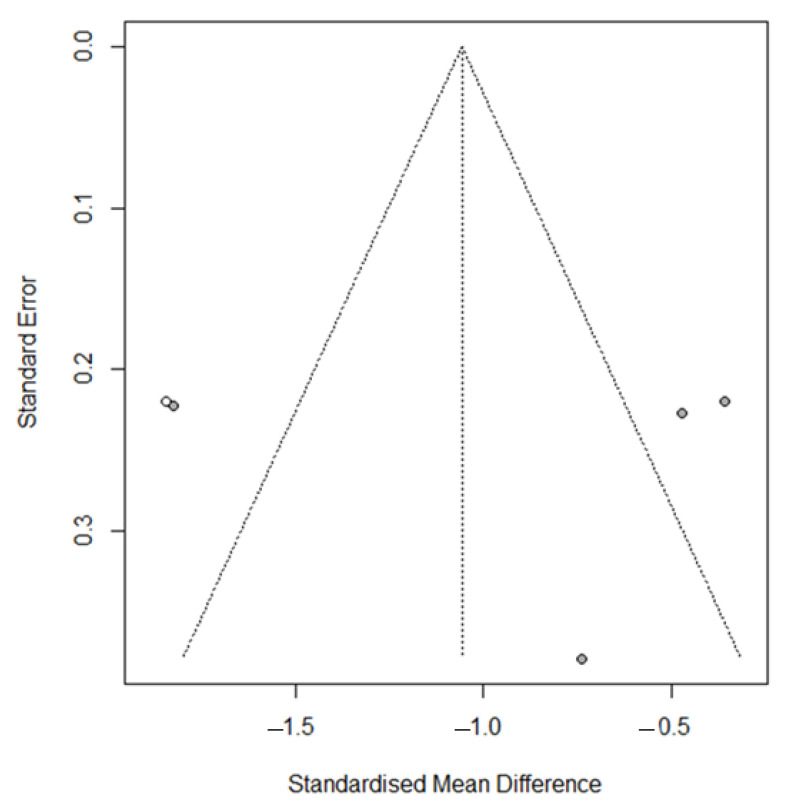
Funnel plot for meta-analysis for evaluation of the NRS/VAS after vitamin D supplementation, after applying the trim and fill method. The filled-in study results are printed as open circles.

Visual inspection of the funnel plot identified asymmetry, confirmed by the trim and fill method, with one added study printed as open circles (*p* = 0.0016) (Figure 3), confirmed by Egger’s test (t = 0.11 *p* = 0.92).

#### 3.4.1. Sensitivity Analyses in NRS/VAS Test

**Figure 4:**
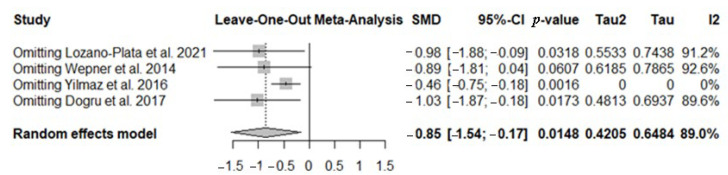
The forest plot identifies the weight of the studies used in the meta-analysis in the NRS/VAS in FMS patients after vitamin D supplementation. SMD, standard mean difference; CI, confidence interval [44,45,46,48].

To further investigate the high heterogeneity observed, a sensitivity analysis was conducted to assess the influence of each individual study on the overall results of the meta-analysis. The findings showed that all included studies had a similar statistical weight, suggesting that no single study disproportionately influenced the overall effect (Figure 4). Indeed, the *leave-one-out* analysis (sequential exclusion of one study at a time) confirmed that the overall effect remained consistent even when these studies were omitted, indicating that the results of the meta-analysis are robust and not critically dependent on individual studies; the only study at the borderline was that of Wepner (Figure 4).

#### 3.4.2. Multivariate Meta-Regression Analysis in NRS Test

The substantial heterogeneity observed in this meta-analysis provided the opportunity to explore several potential confounding variables through meta-regression analysis, including publication year, age, duration of treatment in weeks, and Vitamin D dosage.

**Figure 5:**
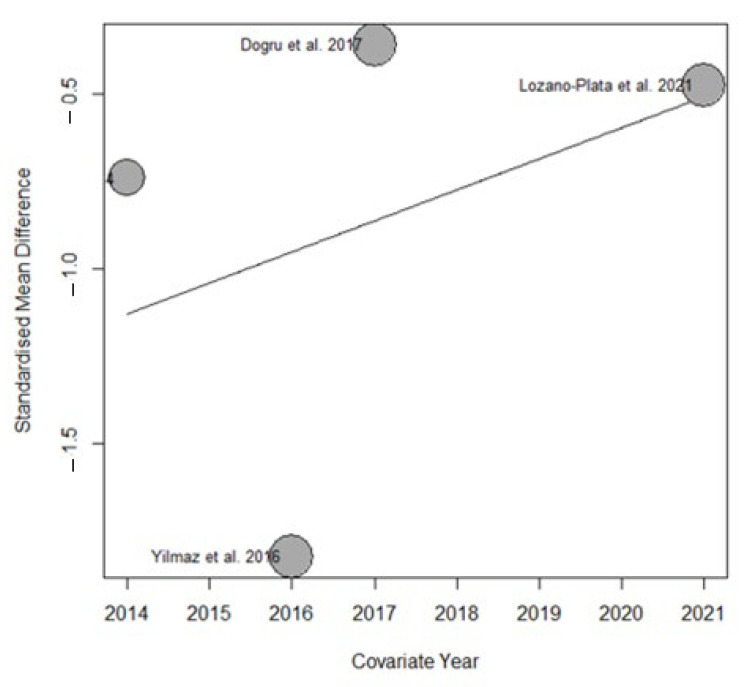
Bubble plot from the meta-regression assessing the effect of publication year on the estimated effect size.

Variables, such as publication year (Figure 5), patient age, duration of treatment in weeks, and Vitamin D dosage, did not result as statistically significant (R^2^ = 0%).

### 3.5. Meta-Analysis and FIQ Scale

**Figure 6:**
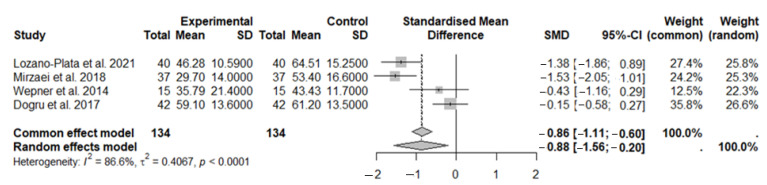
The forest plot identifies an improvement in disease status through the FIQ in FMS patients after vitamin D supplementation. SD, standard deviation; SMD, standard mean difference; CI, confidence interval [44,45,47,48].

In this second meta-analysis, we assessed the improvement in the impact of fibromyalgia on quality of life through the evaluation of the FIQ after months of vitamin D supplementation. A total of 134 subjects were included in the experimental group, and 134 subjects were included in the control group (Figure 6). The results obtained using the random-effects model showed a beneficial effect of vitamin D supplementation in subjects with fibromyalgia symptoms compared to the control group (SMD: −0.88, 95% CI: −1.56 to −0.20; *p* = 0.0115). High heterogeneity was observed among the studies (I^2^ = 86.6%; *p* < 0.0001).

**Figure 7:**
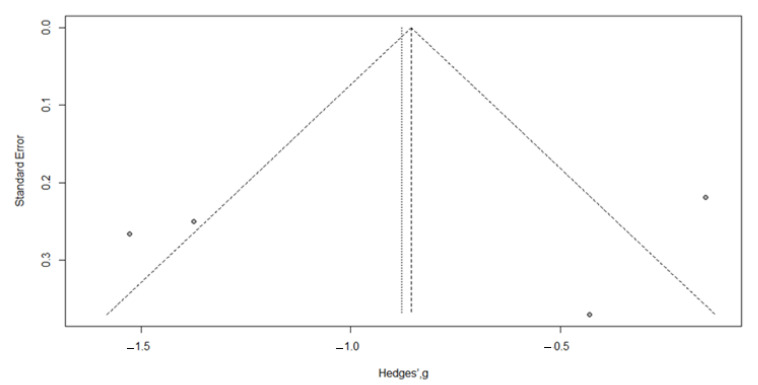
Funnel plot for meta-analysis for evaluation of the FIQ after vitamin D supplementation, after applying the trim and fill method. The filled-in study results are printed as open circles.

Visual inspection of the funnel plot identified a symmetry, confirmed by the trim and fill method, with zero added studies (Figure 7); Egger’s test (t = −0.20, *p* = 0.86).

#### 3.5.1. Sensitivity Analyses in FIQ Test

**Figure 8:**
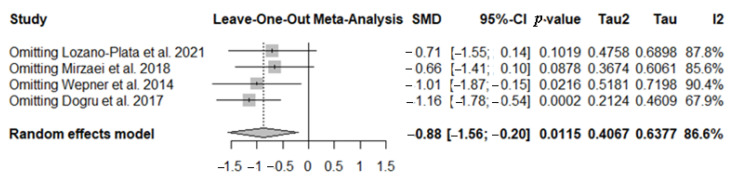
The forest plot identifies the weight of the studies used in the meta-analysis in the FIQ in FMS patients after vitamin D supplementation. SMD, standard mean difference; CI, confidence interval [44,45,47,48].

To further investigate the high heterogeneity observed, a sensitivity analysis was conducted to assess the influence of each individual study on the overall results of the meta-analysis. The *leave-one-out* analysis (sequential exclusion of one study at a time) confirmed that the overall effect remains negative in all cases. However, the studies by Lozano-Plata et al. (2021) [44] and Mirzaei et al. (2018) [47] appear influential, as their exclusion renders the effect non-significant (*p* > 0.05). The study by Wepner et al. (2014) [45] is somewhat borderline, as its exclusion leads to a slightly larger estimated effect and higher I^2^; however, the overall result remains statistically significant. These findings suggest that, although some individual studies affect the magnitude and significance of the effect, the overall meta-analytic finding is robust and consistently negative (Figure 8).

#### 3.5.2. Multivariate Meta-Regression Analysis in FIQ Test

The substantial heterogeneity observed in this meta-analysis provided the opportunity to explore several potential confounding variables through meta-regression analysis, including publication year, age, duration of treatment in weeks, and vitamin D dosage.

Variables, such as patient age, duration of treatment in weeks, and vitamin D dosage, did not show statistically significant results (R^2^ = 0%)

**Figure 9:**
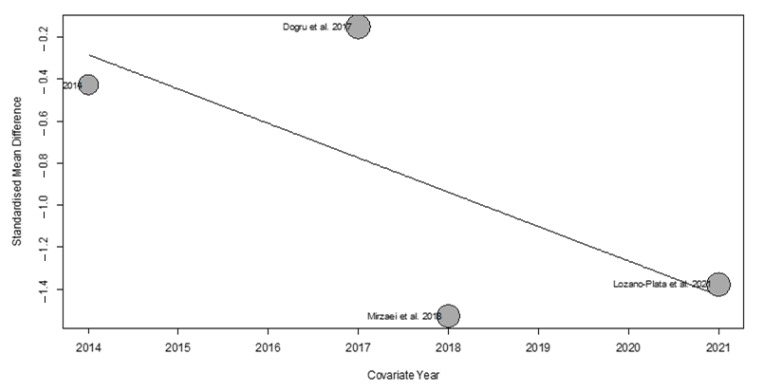
Bubble plot from the meta-regression assessing the effect of publication year on the estimated effect size.

However, we observed that the year of publication partially contributed to the overall heterogeneity of the results. Specifically, the meta-regression analysis indicated that this variable accounted for 19.29% (R^2^ = 19.29%) of the total variability observed across studies. This effect is illustrated in the bubble plot (Figure 9), which highlights the relationship between publication year and the estimated effect size.

## 4. Discussion

FMS is a complex and still not fully understood clinical condition, characterized by a multidimensional symptomatology that includes widespread musculoskeletal pain, chronic fatigue, sleep disturbances, cognitive alterations (the so-called “fibro-fog”) and frequent psychiatric comorbidities such as anxiety and depression [3]. This syndrome, currently classified among central sensitization disorders, is the result of an altered processing of the pain signal at the level of the central nervous system, with consequent pathological amplification of nociceptive perception and reduced pain threshold [49].

The precise etiology remains elusive, although numerous studies have highlighted a frequent onset following traumatic physical events, such as accidents or surgery, or psychological events such as acute or chronic stress [50].

Fibromyalgia has a prevalence of 2–3% globally, with peaks in women aged 20 to 55, where it represents the main cause of musculoskeletal pain [51,52]. Women are twice as likely to be diagnosed as men, and over 40% of patients in specialized pain clinics meet the criteria for fibromyalgia [53]. Risk is increased in those with existing rheumatic diseases.

Juvenile Primary Fibromyalgia Syndrome (JPFP) affects 1–6% of children/adolescents, making up 7–15% of pediatric rheumatology referrals [14,54]. Eighty-four percent of cases occur in girls (mean age 15.4 years), mostly non-Hispanic white [55], but some experts believe that the data are underestimated due to missed diagnoses in ethnic minorities.

The evolution of the terminology from “fibrositis” to “fibromyalgia” reflects the progress in understanding the disease, moving from the original inflammatory hypothesis to the current physiopathological model that emphasizes the neurocentral mechanisms of altered pain processing [56].

“The treatment of fibromyalgia must be based on a multidisciplinary and individualized approach, integrating both non-pharmacological therapies—including personalized physical activity, cognitive-behavioral psychological support and methods for stress control—and traditional treatments based on antidepressants, antiepileptic drugs and muscle relaxants, without neglecting any emerging therapies or innovative supplements” [57].

Among the latter, vitamin D has recently attracted considerable scientific interest because of epidemiological observations that have highlighted a frequent association between hypovitaminosis D and various chronic pain conditions [58]. Numerous studies have shown that vitamin D has anti-inflammatory and immunomodulatory properties [46,59], including downregulation of pro-inflammatory cytokines such as IL-6 and TNF-α and upregulation of anti-inflammatory cytokines such as IL-10 [10]. Additionally, it may modulate pain signaling through the VDR expressed in the central nervous system [60], potentially linking hypovitaminosis D with exacerbation of fibromyalgia symptoms [9,19].

This systematic review and meta-analysis aimed to evaluate the effects of vitamin D supplementation on pain reduction and improvement in quality of life in FMS. Across included studies, the meta-analysis demonstrated that vitamin D significantly reduces pain intensity (NRS/VAS: SMD −0.85; 95% CI −1.54 to −0.17; *p* = 0.0148) and may improve overall quality of life (FIQ: SMD −0.88; 95% CI −1.56 to −0.20; *p* = 0.0115). However, high heterogeneity was observed for both outcomes (I^2^ = 89% for NRS/VAS; I^2^ = 86.6% for FIQ), likely reflecting differences in supplementation protocols, baseline patient characteristics, assessment tools, and study designs. Sensitivity analyses and *leave-one-out tests* confirmed the robustness of the overall effects, although individual studies, particularly Lozano-Plata et al. (2021) [44] and Mirzaei et al. (2018) [47], had an influence on the FIQ outcome. Meta-regression analyses did not identify treatment duration, vitamin D dosage, or patient age as significant contributors to heterogeneity. Publication year partially explained variability in FIQ outcomes, accounting for 19.29% of the total variance, suggesting potential temporal trends in study design or reporting.

### Limitations

This systematic review and meta-analysis present several limitations that must be considered in the results regarding the effectiveness of vitamin D in fibromyalgia.

A significant limitation is the high heterogeneity across studies, as revealed in the statistical analyses. Furthermore, asymmetry in the funnel plot suggests a possible publication bias, likely due to the publication of studies with positive results. However, statistical tests (such as Egger’s test) did not indicate significant evidence of such bias.

A further limitation is the relatively small sample sizes which reduce the statistical power and the robustness of the conclusions.

Despite these limitations, the data show a positive trend in patients treated with vitamin D, with improvements observed in both pain reduction (measured via NRS/VAS) and in the overall impact on the quality of life (assessed using the FIQ).

Further controlled studies are needed to clarify the true therapeutic potential of this supplementation.

## 5. Conclusions

In conclusion, this systematic review and meta-analysis suggest that vitamin D supplementation may contribute to pain reduction in FMS individuals, as indicated by improvements in NRS/VAS scores. Some evidence also suggests a positive impact on overall quality of life (FIQ). However, the high heterogeneity observed across studies for both outcomes (I^2^ = 89% for NRS, I^2^ = 86.6% for FIQ) limits the robustness of these findings. Therefore, while vitamin D could be considered as a complementary strategy within a multimodal treatment approach, these results should be interpreted with caution. Future randomized controlled trials with standardized protocols and investigation of predictive biomarkers are needed to clarify the optimal use and identify patient populations most likely to benefit from vitamin D supplementation. Understanding the molecular mechanisms by which vitamin D modulates pain perception and inflammation, and contributes to a more effective therapeutic-rehabilitative approach for patients, together with the identification of predictive biomarkers of response, represents a promising direction for future research in this field. From a clinical perspective, vitamin D supplementation could be considered a complementary approach within the multimodal management of FMS, particularly in patients with baseline deficiency or in subgroups that may benefit most. Nevertheless, individualized evaluation and careful monitoring remain essential.

## Figures and Tables

**Table 1:** Graphic representation of study design.

CRITERION	INCLUSION	EXCLUSION
**Population**	Human participants with a diagnosis of FMS	Participants with chronic widespread pain (CWP) without a specific FMs diagnosis; animal or in vitro studies
**Intervention**	Vitamin D supplementation	Interventions without vitamin D; vitamin D is not part of the treatment
**Comparison**	Placebo; no treatment; people before treatment	No comparator
**Outcome**	Clinical outcomes for FMS (pain, quality of life)	Incomplete studies
**Study Design**	Interventional studies	Incomplete studies; abstracts, editorials, book chapters
**Language**	Articles published in English	Articles not published in English

## Data Availability

All data generated/analyzed throughout this research are included in this article.
